# Multi-country case studies on planning RMNCH services using WISN methodology: Bangladesh, Ghana, Kenya, Sultanate of Oman and Papua New Guinea

**DOI:** 10.1186/s12960-021-00671-3

**Published:** 2022-01-28

**Authors:** Teena Kunjumen, Mollent Okech, Deki, James Avoka Asamani, Nazar Mohamed, Md. Nuruzzaman

**Affiliations:** 1 World Health Organisation, Geneva, Switzerland; 2 World Health Organisation, Port Moresby, Papua New Guinea; 3 World Health Organisation, Suva, Fiji; 4 World Health Organisation, Harare, Zimbabwe; 5 Ministry of Health, Muscat, Sultanate of Oman; 6 World Health Organisation, Dhaka, Bangladesh

**Keywords:** Workload components, Activity standards, Reproductive, Maternal, Newborn, Child, Primary care, Workload indicators of staffing need

## Abstract

**Background:**

Globally, many countries are adopting evidence-based workforce planning that facilitates progress towards achieving sustainable development goals for reproductive, maternal newborn and child health. We reviewed case studies on workforce planning for reproductive maternal newborn child health services at primary care level facilities using workload indicators of staffing need in five countries.

**Method:**

Using available workload indicators for staffing need reports from Bangladesh, Ghana, Kenya, Sultanate of Oman and Papua New Guinea, we generated descriptive statistics to explore comparable workload components and activity standards, health service delivery models with an emphasis on the primary care levels and the specific health occupations offering interventions associated with reproductive maternal, newborn and child health services.

**Results:**

The health services delivery models vary from one country to another. The results showed variability in the countries, in the workload components and activity standards of each regardless of facility level or occupational groups involved. All the countries have decentralized health services with emphasis on comprehensive primary care. Reproductive, maternal and new-born child health care services include antenatal, postnatal, immunization, family planning, baby wellness clinics, delivery and management of integrated minor childhood illnesses. Only Sultanate of Oman offers fertility services at primary care. Kenya has expanded interventions in the households and communities.

**Conclusion:**

Since the health care services models, health services delivery contexts and the health care worker teams vary from one country to another, the study therefore concludes that activity standards cannot be adopted or adapted from one country to another despite having similar workload components. Evidence based workforce planning must be context-specific, and therefore requires that each country develop its own workload components and activity standards aligned to their local contexts.

## Background

Health workforce policies based on sound evidence is essential to ensure the provision of quality health services and to achieve the national set targets of universal health coverage (UHC) and sustainable development goals (SDGs) [1, 2]. There is an accumulating body of evidence that increased availability of skilled health workforce is linked to improved health outcomes including reproductive, maternal, newborn and child health (RMNCH) [3, 4]. However, there are significant variations in the availability, distribution and utilization of primary health care workers, services provided and time taken to undertake any given intervention [5]. This paper focuses on evidence-based workforce planning using workload components and activity standards [6] at the different levels of care for the specific health occupations to achieve RMNCH services in five countries.

The Nairobi Summit on ICPD25 held in November 2019 brought together over 8300 delegates from about 170 countries and partners who made bold commitments to transform the world by ending all maternal deaths, unmet need for family planning and gender-based violence and harmful practices against women and girls by 2030 [7]. The meeting aimed at renewing, re-energizing communities to work together to support strengthening of national health systems in order to enable accelerated delivery of key interventions for improved RMNCH outcomes along the continuum of care. This cannot be achieved without well planned adequate healthcare providers.

Primary health care (PHC) has been proven to be a highly effective and efficient way to address the leading causes and risks of poor health and well-being today, as well as handling the emerging challenges that threaten health and well-being tomorrow. It has also been shown to be a good value investment, as there is evidence that quality primary health care reduces total healthcare costs and improves efficiency [8]. Countries that adopt health systems with strong emphasis on strengthening the primary care levels are more likely to have better and more equitable outcomes due to being more efficient and having lower costs for essential health services [9]. This in turn, leads to improved client satisfaction than those whose systems have only a weak orientation to PHC. Universal access to reproductive, maternal, newborn and child health is one of the key components of the sustainable development goals (SDGs) [10]. The World Health Organization (WHO) and other health partners continue to support countries to strengthen health systems and to cultivate enabling environments where skilled health professionals provide quality reproductive, maternal, newborn and child health services [11]

In countries like Bangladesh, Ghana, Kenya, Sultanate of Oman and Papua New Guinea, primary care is emphasized as a strategic priority for increasing access to essential services including RMNCH services [12–16]. As countries work towards achieving UHC and SDGs, discussions relating to evidence based workforce planning, deployment and management are progressively getting attention in many countries’ policies. Human resources for health are increasingly considered an important change factor and highly relevant to economic development [17].

The Global Strategy for Women’s, Children’s and Adolescents’ Health (2016–2030) [18] envisions a world in which every woman, child and adolescent in every setting realizes their rights to physical and mental health and well-being, has social and economic opportunities, and is able to participate fully in shaping prosperous and sustainable societies. It provides a significant opportunity to broaden government’s actions to commit financing and improve universal access to sexual and reproductive health rights as part of the universal health coverage and sustain gains made drawing on demographic diversity to drive economic growth. The Global Strategy on Human Resources for Health (GSHRH) estimates a minimum threshold of 4.45 physicians, nurses and midwives per 1000 population as necessary for making sustained progress towards the SDGs and other health goals for meeting the health needs of a country. With many primary health care models and health occupations worldwide, increased attention is being focused on human resources planning and management. Specifically, human resources are one of three principle health system inputs, with the other two major inputs being physical capital and consumables. To achieve a reasonably sized health workforce that can meet the population needs, countries, need to shape national and subnational level policies and relevant health goals [5]. Although WISN is the most methodologically consistent and widely used evidence-based health workforce planning tool, cross-country comparisons of the service standards and contexts that drive WISN-based workforce requirements have not been scientifically documented.

This paper examines how evidence-based health workforce needs were estimated for reproductive, maternal newborn child health (RMNCH) services at primary health care level health facilities in Bangladesh, Ghana, Kenya, Sultanate of Oman and Papua New Guinea using the WISN method.

## Methods

To describe the five countries’ workload components, we purposively narrowed down to those countries that had used the workload indicators of staffing need (WISN) methodology to generate evidence enabling policy changes or new initiatives in the country. We focused on primary health care facilities in service areas that offer RMNCH interventions. A total of 8 RMNCH interventions were identified, assessed and selected for this review with the specific health worker occupations that performed the interventions. Key variables chosen for this study included the workload components and activity or service standards for health service activities. This was a comparative study that involved five countries—Bangladesh, Ghana, Kenya, Sultanate of Oman and Papua New Guinea.

In each country under study, the WISN implementation was undertaken by three committees namely the Steering Committee, technical task force (TTF) and the Expert Working Groups (EWG). The committees had specific roles in the WISN process to ensure accurate implementation. The committee members were oriented with the support of WHO and other partners. Each country’s expert working groups (EWG) independently developed workload components and activity standards within their local contexts under the guidance of technical task force. The countries in the study focused on all the interventions undertaken in the health facilities however; our current study only discusses selected RMNCH services.

Cross-country analysis followed core principles of qualitative data analysis. The authors thoroughly reviewed all country reports to pick out the RMNCH services offered at primary care facilities. Thematic analysis was then completed to identify common services, occupations performing them, and the time taken to undertake the workload components.

## Results

### Health service delivery models across countries

The delivery models and services packages differ from one country to another with different health workers and services for the essential RMNCH services. Papua New Guinea’s governance system is highly decentralized with each of the provinces responsible for delivery of health and other social services supported by legislative laws. There are seven levels of PNG’s health service delivery model as outlined by the National Health Services Standards (NHSS): aid posts, sub-health centres/community health posts, health centres, district hospitals, provincial hospitals, regional hospitals and the national referral hospitals.

Kenya’s healthcare system is decentralized with each of the counties responsible for health services. The community unit is the foundation of the service delivery system with both demand creation and health promotion services. The physical level of the health system is the dispensary and health centre. The County referral health services comprise all level 4 and level 5 secondary hospitals and services in the country. Finally, the national referral services comprise all tertiary level 6 referral hospitals, national reference laboratories and services, blood transfusion services, research and training institutions providing highly specialized care.

The health service delivery system in the Sultanate of Oman is divided into three levels: primary, secondary, and tertiary. The primary level comprises of health centers and extended health centers (polyclinics), secondary level to district and regional hospitals, and tertiary level to the four national hospitals. Within this system, the Ministry of Health (MoH) provides more than 80% of the health services.

Bangladesh has an extensive healthcare delivery network from the central to the rural communities. The network is divided into three levels of care i.e. primary, secondary and tertiary levels. The primary level includes health facilities built at wards (household/community) level, Union (several wards constitute a Union) level and Upazila or sub-district level (several Unions constitute an Upazila). Secondary level includes health facilities built at district cities and tertiary level facilities are built at district, divisional and capital cities.

Ghana is administratively divided into regions, districts, sub-districts and communities. The delivery of health services is operationally and administratively aligned with these structures. The public health service delivery is entrusted to semi-autonomous establishments, namely, the Ghana Health Service (GHS) for primary levels of care and Teachings Hospitals (THs) for tertiary and quaternary levels of care.

Table 1 highlights primary health care facilities in the five countries, while the explanations are provided in Appendix (Tables 4, 5, 6, 7, 8).

**Table 1:** Health facilities at the PHC level in the five countries

Primary health care levels	Bangladesh	Ghana	Kenya	Oman	Papua New Guinea
Level 1	Community clinic	Community-based health planning and services (CHPS) Compound/zones	Community unit	Health centre/Polyclinics (extended health centres)	Aid post
Level 2	Union Health and Family Welfare Center, Union Sub Center	Health centre	Dispensary	-NA-	Community health post/sub centre
Level 3	Upazila /Sub-district Health	District Hospital	Health centre	-NA-	Health centres
Level 4	-NA-	-NA-	-NA-	-NA-	District hospitals

### Analysing the workload components and activity standards for RMNCH services using WISN

In implementing WISN, one of the key steps is the definition of the health occupation activities that make up their daily schedule, which are termed as the workload components. The process of establishing health service activities and the activity standards in the five countries was done by the EWG based on their experiences in the health facilities and their competencies. Typically, workloads are categorised into three different workload components namely health service activities, support activities and additional activities. This multi-country study focuses on health service activities and their corresponding activity/service standards. Health service activities are all those tasks performed by all members of that staff category (an occupational group) based on their training and competencies. They have regular statistics collected on them. They involve direct provision of health services to patients or clients. Using this definition, we synthesized the workload components and activity standards for RMNCH services at the primary health care level for the five countries which cover pre-natal, pregnancy, childbirth, postnatal and childhood. Table 2 provides a summary of the RMNCH services provided at PHC level across the five countries. Further details can be found in Appendix (Tables 9, 10, 11, 12 and 13).

**Table 2:** RMNCH services provided at PHC level in the five countries

Standardized level of care facility	Standardized workload component (Health activity)	Bangladesh	Ghana	Kenya	Oman	Papua New Guinea
Level 1	ANC				45	15
	Baby wellness clinics		10		25	15
	Family planning		75	46	35	15
	First ANC	20				
	Follow up ANC	10	15			
	Immunization	47	5		30	10
	Infertility treatment				50	
	PNC	15	15		45	15
Level 2	ANC					15
	Baby wellness clinics		15	49		15
	Family planning		75	46		15
	First ANC	20	25	41		
	Follow up ANC	10	15	16		
	Immunization		5	34		10
	PNC	15	15	28		15
Level 3	ANC					15
	Baby wellness clinics	35	15	49		15
	Family planning	55	75	46		15
	First ANC	20	25	41		
	Follow up ANC	10	15	16		
	Immunization		5	34		10
	PNC	15	15	28		15
Level 4	ANC					15
	Baby wellness clinics					15
	Family planning					15
	Immunization					10
	PNC					15

The results show that the five countries plan and provide these essential services differently. The results also showed differences in the countries, in the workload components and activity standards of each facility level or occupational groups involved. In Papua New Guinea health workers at all the primary care services offer ANC services for 15 min per client. In Sultanate of Oman ANC services are offered for 45 min per client, 25 min by medical doctors and 20 min by nurses. Either of the two can offer the services at any one time. Papua New Guinea and Sultanate of Oman thus plan for the same time for ANC services. Kenya estimates more time for the 1st ANC at 35 min per client and 15 min for the subsequent ANC visits. In Ghana, health workers also spent 25 min per client on the first ANC visit and 15 min for subsequent ones. In Bangladesh, health workers also spent 60 min per client on the first ANC visit and 30 min for subsequent ones. Out of the five countries under review only Sultanate of Oman provided infertility treatments as part of PHC services.

Primary care level facilities in Papua New Guinea offer RMNCH services in the aid posts, sub health centers, health centers and district hospitals. The services are provided by community health workers, nursing officers and the midwives. The services can be provided by any of the occupations at any one time. Kenya on the other hand offers primary care services in the community health units outside the health facilities, in the dispensaries and in the health centres. The types of care offered at the different levels of primary care differ based on the service and the cadres offering them at each level.

The services can be provided by any of the occupations at any one time. Sultanate of Oman offers the primary health care services in the health centres and polyclinics, where services are provided by nurses and the general medical practitioners simultaneously. Bangladesh offers primary care health services in the community clinics, union sub centers, union health family welfare clinics and upazila health complex. The services are offered by health care providers, midwives, family welfare visitors, sub assistant community medical officers, staff nurses and medical officers. Ghana offers PHC services in community-based health planning and services (CHPS) compound, health centres and district hospitals. The services are delivered by community health nurses and midwives. Appendix (Tables 9, 10, 11, 12 and 13) shows RMNCH services, the times taken and the specific occupations offering them in the five countries. Further, the health occupations that provide the RMNCH services at PHC level were mapped according to the International Standard Classification of Occupations (ISCO-08) [19] as shown in Table 3.

**Table 3:** Health occupations involved in RMNCH services at PHC level in the five countries

Health occupation titles (ISCO-08)	Bangladesh	Ghana	Kenya	Oman	Papua New Guinea
Generalist medical practitioners (2211)	Medical Officer		Medical Officer	Doctor (GP)	
Nurses (2221, 3221)	Staff Nurse	Community Health Nurse (CHN)	Nurse	Nurse	Nursing officer
Midwives (2222, 3222)	Midwife	Midwife			Midwife
Paramedical practitioners (2240)	Sub-Assistant Community Medical Officer (SACMO)		Clinical officer		
Community health workers (3233)	Family welfare visitor Community Health Care Provider (CHCP) Health Assistant		Community health assistant (CHA), Community health officer (CHO), Community health volunteer (CHV)		Community health worker (CHW)

We also examined how the health workers in the primary health care facilities distributed their health service activities time for RMNCH services. This enables the understanding of how these services are offered along the continuum of care from pre-natal through pregnancy, childbirth, post-natal and to early childhood. Priority interventions show the linkages and help in contextualising services provided in the consecutive life stages. Bangladesh, Ghana and Kenya record first ANC and follow up ANC separately with different times while Oman and Papua New Guinea do not differentiate the first ANC and the follow up ANC.

**Fig. 1:**
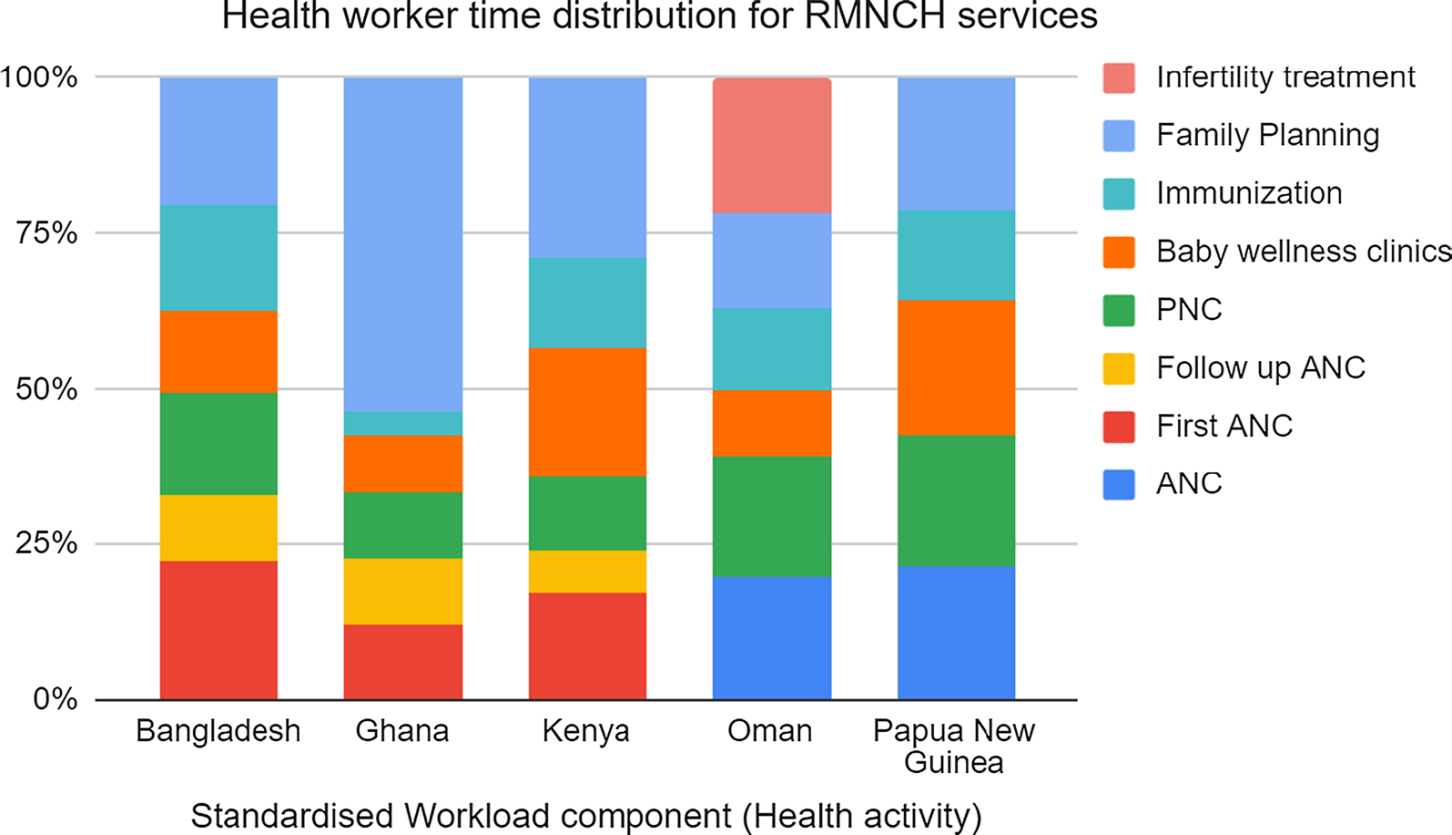
Health worker time distribution for RMNCH services in the five countries

Using the total time dedicated to RMNCH activities as a denominator, we analyzed the time distribution across the RMNCH services. It revealed that a substantial proportion of the health workers time is spent on offering ANC and PNC services. At the PHC level, 50% of health workers time in Bangladesh, 42% in Papua New Guinea, 40% in the Sultanate of Oman, 36% in Kenya and 33% of the health workers time in Ghana is spent on ANC and PNC interventions. Further details on baby wellness, immunizations and family planning show that countries dedicated different times for the services. Ghana primary health care workers dedicated over 50% of their time to offer family planning services, while Kenyan primary health care workers dedicated 30% of their time to the same family planning, Bangladesh and Papua New Guinea primary health care workers both dedicated 20% of their time to family planning and only 15% was dedicated for family planning by the primary health care workers of the Sultanate of Oman. Papua New Guinea health workers spent an average of 35% for child health in immunizations and baby wellness, Kenya and Bangladesh spent 30% while Ghana and Sultanate of Oman spent 15% each for immunizations and baby wellness interventions. Only Sultanate of Oman provided infertility treatment at primary care level facilities and the health care workers dedicated 21% of their time to offer this service. None of the countries had similar time distribution for RMNCH services as shown in Fig. 1.

## Discussion

This his paper focused on a review and synthesis of selected health services related to RMNCH health interventions offered at the primary health care facilities of five countries. The activities take up most of a health worker’s time and have annual statistics regularly collected on them. Also considered in the review was the amount of time necessary for a well-trained skilled and motivated worker to perform these activities to their professional standards in the given circumstances of the countries [20]. These variables helped in assessing the length of professional time for each of the occupations required to execute the RMNCH services.

Increasingly, across the globe, there is emphasis on establishing integrated service delivery through the continuum of care’. This system provides a range of health services in the various life stages including the adolescence, pre-natal, childbirth and postnatal period, childhood and through reproductive age so that care can evolve with the patient over time [21, 22]. Integrated services enhance access to services by ensuring coordinated care. For example a mother can schedule to have her family planning visits together with immunizations at the same time thus benefits are extended to the patients, caregivers and the larger health care systems.

In addition, services should be available at all levels including in homes and communities, through outpatient services and hospitals with ‘inpatient’ facilities to clearly connect the services to referral-level services. This approach is based on the sound principle that the health of an individual across the life stages is interlinked [23]. The time health workers take to offer services is also an important factor. Although the principle of equal access to medically justified treatment with the standard times has been promoted by official health policies and guidelines, practices do not completely meet these targets. Good health of both the mother and the newborn are directly linked and any health intervention to prevent mortality applies to both the mother and newborn. There is evidence that simple and timely intervention like antenatal care, skilled childbirth, post-natal and new-born care can bring about positive impact on RMNCH services [6, 24].

Examining case studies makes it evident that human resources planning based on workload components and activity standards can and does play an essential role in the primary health care system. The practices, policies and relevant competencies of health occupations are imperative in developing and improving reproductive, maternal, newborn and child health services. The implication is that further research and studies must be conducted in order to determine additional health service interventions that can be beneficial to the health sector.

## Limitations

First, RMNCH services remain poorly documented in electronic databases; it may therefore be possible that, despite the very careful and extensive selection and expert consultations, some relevant RMNCH interventions were not identified. Second, there are no widely accepted models of primary health care delivery models and health occupations. Though we have carefully specified the inclusion criteria, there is a need for a clear taxonomy of primary health care occupations. Nevertheless, key data were available and rigorous methods have been applied throughout, including the risk of bias assessments and evaluation of the quality of the evidence.

## Conclusion

This comparative multi-country case study on workload components and activity standards for RMNCH serves as a configuration for health teams. This study concludes that the health care service models, health services delivery contexts, health care worker teams, workload components and their sub activities vary from one country to another. It will be therefore inappropriate to develop activity standards to be adopted or adapted from one country to another despite the seeming similarity in the defined workload components and their related sub components for RMNCH at the PHC level. Evidence based workforce planning therefore requires that each country develops its own workload components, its sub activities and activity standards aligned to their own local contexts.

## Data Availability

All data generated or analysed during this study are included in this published article. Information supporting the data can be found in the complete WISN report for the various countries.

## Appendix

See below Tables 4, 5, 6, 7, 8, 9, 10, 11, 12 and 13.

**Table 4:** Service delivery model in Papua New Guinea

Level of care	Services offered	Occupations involved
Level 1: Aid post Serves 1000 to 2000 population	Health promotion, health improvement, health protection, primary health and maternity care at community level through an established referral pathway	Community health workers
Level 2: community health post/sub centre Serves 4000 to 10 000	Inpatient short stay care, integration of outreach/mobile services for family and reproductive health, child health, TB DOTs, HIV/AIDs prevention, malaria prevention, and nutrition programs and school and dental health	Nurses, Community health workers, Pharmacy assistants
Level 3: Health centres Serves 15 000 to 30 000	Minimum level of medical, child health, maternal health, minor surgical services, inpatient care, outreach services and supervision programs	Health extension officers (HEO), nurses, midwives, CHWs, rural laboratory assistant, dental therapist, radiographer and pharmacy technician or pharmacy assistant
Level 4: district hospitals Serves 40 000 to 60 000 people	Medical, child health, maternal health and minor surgical services, inpatient care, outreach services, elective surgeries and supervision programs	Medical officer (rural medicine), health extension officers (HEO), nurses, midwives, CHWs, rural laboratory assistant, dental therapist, radiographer and pharmacy technician or pharmacy assistant

**Table 5:** Service delivery model in Kenya

Level of care	Services offered	Occupations
Level 1: Community Serves less than 5000 population	Mental health, communicable and non-communicable diseases, basic curative services, home based care for terminally ill patients, environmental health services, nutrition services, child health and immunization, New-born care, reproductive health, selected family planning services, behaviour change and communication, services for Orphans and vulnerable groups, gender based violence	Community health assistant (CHA) Community health officer (CHO) Community health volunteer (CHVs)
Level 2: Dispensaries and clinics Serves about 10 000 population	Outpatient services, VCT services, TB services, laboratory services, well baby clinics, family planning services, ANC, PNC, pharmacy services, counselling, curative treatment and referrals	Clinical officer Nurse Laboratory technician
Level 3: Health centres Serves an average of 30 000 population	Maternity in patient, curative services, laboratory services, dental services, counselling, pharmacy, TB clinics, Diabetes, hypertension clinics, CCC for HIV services, well baby clinics, family planning services ANC, PNC, referral services	Clinical officer, nurse, Laboratory technologist, pharmacy technologist

**Table 6:** Service delivery model in Bangladesh

Level of care	Services offered	Occupations
Level 1: Community clinic Serves 6000 population	Outdoor services, maternal and child health care, family planning services, referral, immunization, health education, NCD screening e.g. diabetes check and hypertension	Community Health Care provider (CHCP), Health Assistant (HA), Family Welfare Assistant (FWA)
Level 2: Union Health and Family Welfare Center (UHFWC), Union Sub Center (USC) Serves 40 000 population	Outdoor services; Maternal and child health care; Family planning services, referral	Medical Officer, Sub-Assistant Community Medical Officer (SACMO), Pharmacist (Diploma), Midwife, Family welfare visitor (FWV)
Level 3: Upazila/Sub-district Health Complex Serves 200 000–400 000 population	Indoor and outdoor services including family planning, Maternal and child health care, Orthopaedic cares, Minor surgical services, Major consultant/specialized services, caesarean section, Counselling, health education, immunization, laboratory, NCD management	Consultants, Medical Officer, Dental Surgeon, SACMO, Staff Nurse, Midwife, Pharmacist (Diploma), Medical Technologist

**Table 7:** Service delivery model in Sultanate of Oman

Level of care	Services offered	Occupation
Level 1: Health centres Serves 10 000 population	Outpatient clinics; vaccination and growth monitoring for children; antenatal and postnatal care; family planning, infertility clinics; care of elderly; health promotion and care in communities, schools; mental health; non-communicable; dental and oral health care; laboratory services; dispensing of drugs; preventive care; ambulance services	General medical practitioner, Nurse
Level 1: Polyclinics/extended health centres Serves 15 000–25 000 population	Advance services such as speciality clinics for non-communicable diseases; dermatology; internal medicine; obstetrics and gynaecology; ophthalmology and eye care; ENT; advance screening services; ultrasonography; acute care; advance laboratory investigations; and teaching of residents	General medical practitioner, Nurse

**Table 8:** Service delivery model in Ghana

Level of care	Services offered	Occupations
Level 1: community-based health planning and services (CHPS) Compound/zones Serves 5000 people or 750 households	Preventive care and curative care, maternal and reproductive health, supervised delivery, neonatal and child health services, health education, sanitation and counselling on healthy lifestyles and good nutrition, patient follow-up	Community health nurse, Enrolled Nurse and Midwife
Level 2: Health centres (HCs) Serve 20 000 or fewer people (Urban areas with higher populations have polyclinics)	Preventive care and curative care, outpatient services for communicable and non-communicable diseases e.g. TB. HIV, malaria, hypertension, diabetes etc., maternal services, basic emergency care, referral services	Physician Assistant (Medical), General Nurses, Enrolled Nurses, Midwives Community health nurse, Mental Health Nurse, Laboratory Laboratory Technician, Dispensing Technician and Assistant
Level 3: district/primary hospitals Serves between 100 000 and 200 000 people	Preventive care and curative care, quality clinical care, selected surgeries, laboratory and other diagnostic services, basic inpatient care, training and technical supervision to health centres, non-clinical support services, referral services	Doctors, nurses, midwives, pharmacists, laboratory scientists, and other paramedics

**Table 9:** PHC RMNCH services, the time taken and occupations providing them in Papua New Guinea

Facility	Cadre	Workload component	Activity/service standards
Aid post (level 1)	Community health worker	Antenatal care	15 min/client
		Postnatal care	15 min/client
		Family planning	15 min/client
		Immunization	10 min/patient
		Well baby clinic	15 min/patient
Sub-health center/(level 2)	Nursing Officer, Community Health Worker	Antenatal care	15 min/client
		Postnatal care	15 min/client
		Family planning	15 min/client
		Immunization	10 min/patient
		Well baby clinic	15 min/patient
Health center (level 3)	Nursing Officer, Community Health Worker	Antenatal care	15 min/patient
		Postnatal care	15 min/patient
		Family planning	15 min/patient
		Immunization	10 min/patient
		Well baby clinic	15 min/patient
District hospital (level 4)	Midwife, Nursing Officer, Community Health Worker	Antenatal care	15 min/patient
		Postnatal care	15 min/patient
		Family planning	15 min/patient
		Immunization	10 min/patient
		Well baby clinic	15 min/patient

**Table 10:** PHC RMNCH services, the time taken and occupations providing them in Kenya

Facility	Cadre	Workload component	Activity/service standards
Community unit	Community health assistants, Community health officers, Community health volunteers	Oral immunization	10 min/patient
		Well baby clinic	30 min/client
		IMCI	45 min/client
		Referral	10 min/client
		Male condoms	10 min/client
		Female condoms	13 min/client
		Combined oral contraceptives	12 min/client
		Progestogen only pills (PoPs)	12 min/client
		Natural family planning	15 min/client
Level 2	Nurse, clinical officer	1st ANC booking	35 min/client
		PMTCT	45 min/client
		ANC follow up	35 min/client
		Admission of pregnant women	45 min/client
		Labour management	294 min/client
		Normal delivery	40 min/client
		Immediate newborn care	20 min/client
		Immediate maternal care	15 min/client
		Care newborn after 24 h of birth	40 min/client
		Discharge of mother and child	12 min/client
Level 3—health centers	Nurse, clinical officer, medical officer	Oral immunization	10 min/client
		Injectable immunization	20 min/client
		Well baby clinic	30 min/client
		IMCI	45 min/ patient
		PNC visits	20 min/client
		Referral	10 min/client
		Male condoms	10 min/client
		Female condoms	13 min/client
		Combined oral contraceptives	12 min/client
		Progestogen only pills (PoPs)	12 min/client
		Progestogen only injectable	15 min/client
		Combined injectable contraceptives	15 min/client
		IUD copper	30 min/client
		Natural methods	15 min/client
		HCG/pregnancy	6 min/client

**Table 11:** PHC RMNCH services, the time taken and occupations providing them in Sultanate of Oman

Facility	Cadre	Workload component	Activity/service standards
Level 1—Health centre	General medical practitioners, Nurses	ANC	25 min/client
		PNC	25 min/client
		IMCI	10 min/client
		Immunization	10 min/patient
		Family planning	15 min/client
		Infertility	30 min/client
		ANC	20 min/client
		PNC	20 min/patient
		Immunization	20 min/patient
		Family planning	20 min/patient
		Infertility	20 min/patient
		IMCI	15 min/patient

**Table 12:** PHC RMNCH services, the time taken and occupations providing them in Bangladesh

Facility	Cadre	Workload component	Activity/service standards
Level 1—Community Clinic	Family welfare visitor, Community Health Care Provider, Sub-Assistant Community Medical Officer (SACMO), Health Assistant	First ANC	20 min/client
		Follow up ANC	10 min/client
		Immunization	47 min/patient
		PNC	15 min/client
		IMCI	15 min/patient
Level 2—Union Sub Center (USC) Union Health and Family Welfare Center (UHFWC)	Family welfare visitor, Community Health Care Provider, Sub-Assistant Community Medical Officer (SACMO)	First ANC	20 min/client
		Follow up ANC	10 min/client
		PNC	15 min/patient
Level 3—Upazila Health Complex	Medical Officer, Staff Nurse, Midwife, Family welfare visitor, Sub-Assistant Community Medical Officer (SACMO)	Baby wellness clinics	35 min/client
		Family planning	55 min/client
		First ANC	20 min/patient
		Follow up ANC	10 min /client
		PNC	15 min/patient

**Table 13:** PHC RMNCH services, the time taken and occupations providing them in Ghana

Facility	Cadre	Workload component	Activity/service standards
Level 1—community-based health planning and services (CHPS) Compound	Community health nurse, Midwife	Baby wellness clinics	10 min/client
		Family planning	75 min/client
		Follow up ANC	15 min/client
		Immunization	5 min/client
		PNC	15 min/client
Level 2—Health centre	Community health nurse, Midwife	Baby wellness clinics	15 min/client
		Family planning	75 min/client
		First ANC	25 min/client
		Follow up ANC	15 min/client
		Immunization	5 min/client
		PNC	15 min/client
Level 3—district/primary hospitals	Community health nurse, Midwife	Baby wellness clinics	15 min/client
		Family planning	75 min/client
		First ANC	25 min/client
		Follow up ANC	15 min/client
		Immunization	5 min/client
		PNC	15 min/client

## Abbreviations

ANC: Antenatal care
AWT: Available working time
AS: Activity standards
CHA: Community health assistant
CHCP: Community Health Care Provider
CHN: Community health nurse
CHO: Community health officer
CHV: Community health volunteer
CHW: Community health workers
FWA: Family Welfare Assistant
FWV: Family welfare visitor
HA: Health Assistant
HEO: Health extension officers
PHC: Primary health care
PNC: Postnatal care
RMNCH: Reproductive, maternal and child health
SACMO: Sub-Assistant Community Medical Officer
TTF: Technical task force
UHC: Universal health care
WISN: Workload indicators of staffing need
WHO: World Health Organization
